# Use of cost-effectiveness analysis to compare the efficiency of study identification methods in systematic reviews

**DOI:** 10.1186/s13643-016-0315-4

**Published:** 2016-08-17

**Authors:** Ian Shemilt, Nada Khan, Sophie Park, James Thomas

**Affiliations:** 1 Social Sciences Research Unit, UCL Institute of Education, London, UK; 2 Department of Primary Care and Population Health, University College London, London, UK

## Abstract

**Background:**

Meta-research studies investigating methods, systems, and processes designed to improve the efficiency of systematic review workflows can contribute to building an evidence base that can help to increase value and reduce waste in research. This study demonstrates the use of an economic evaluation framework to compare the costs and effects of four variant approaches to identifying eligible studies for consideration in systematic reviews.

**Methods:**

A cost-effectiveness analysis was conducted using a basic decision-analytic model, to compare the relative efficiency of ‘safety first’, ‘double screening’, ‘single screening’ and ‘single screening with text mining’ approaches in the title-abstract screening stage of a ‘case study’ systematic review about undergraduate medical education in UK general practice settings. Incremental cost-effectiveness ratios (ICERs) were calculated as the ‘incremental cost per citation ‘saved’ from inappropriate exclusion’ from the review. Resource use and effect parameters were estimated based on retrospective analysis of ‘review process’ meta-data curated alongside the ‘case study’ review, in conjunction with retrospective simulation studies to model the integrated use of text mining. Unit cost parameters were estimated based on the ‘case study’ review’s project budget. A base case analysis was conducted, with deterministic sensitivity analyses to investigate the impact of variations in values of key parameters.

**Results:**

Use of ‘single screening with text mining’ would have resulted in title-abstract screening workload reductions (base case analysis) of >60 % compared with other approaches. Across modelled scenarios, the ‘safety first’ approach was, consistently, equally effective and less costly than conventional ‘double screening’. Compared with ‘single screening with text mining’, estimated ICERs for the two non-dominated approaches (base case analyses) ranged from £1975 (‘single screening’ *without* a ‘provisionally included’ code) to £4427 (‘safety first’ *with* a ‘provisionally included’ code) per citation ‘saved’. Patterns of results were consistent between base case and sensitivity analyses.

**Conclusions:**

Alternatives to the conventional ‘double screening’ approach, integrating text mining, warrant further consideration as potentially more efficient approaches to identifying eligible studies for systematic reviews. Comparable economic evaluations conducted using other systematic review datasets are needed to determine the generalisability of these findings and to build an evidence base to inform guidance for review authors.

## Background

A series of recent journal articles highlighted the urgent need for more efficient prioritisation, design, conduct, analysis, management and regulation of research in order to increase its value and reduce waste, with the goal of improving the ways study data are curated, synthesised, used and re-used to inform decision-making about health and well-being [1–5]. It is therefore important to evaluate the costs and effects of methods, systems and processes designed to improve the efficiency of systematic review and evidence synthesis production workflows.

Economic evaluations are comparative analyses that assess alternative courses of action in terms of both their costs and effects and can be used to evaluate alternative methods, systems and processes. Study data compiled from economic evaluations conducted as ‘meta-research’ (‘research on research’) [6, 7] can build into an evidence base for use to inform, for example: (i) *decisions about the adoption of new methods* proposed as adjuncts to, or replacements for, those commonly applied to achieve a given output at a given procedural stage of a systematic review or evidence synthesis workflow and/or (ii) *choices between existing methods* that could, in principle, each be applied to achieve the same output at a given stage of such workflows. With evidence from well-conducted economic evaluations in hand, decisions and choices about methods can be made on grounds of efficiency.

In this article, we aim to demonstrate the application of an economic evaluation framework to compare the costs and effects of four (*x*2) variant approaches to identifying studies for inclusion in systematic reviews. This evaluation framework is transferable and can be flexibly implemented by other systematic review authors as a ‘*S*tudy *W*ithin *A R*eview’ (SWAR) [8], in order to help build an evidence base to underpin updated guidance for systematic review authors on study identification methods (for example, [9–11]). In the context of this evidence base, the current ‘case study’ can be viewed as an ‘*n* of 1’ study that contributes a single SWAR dataset for potential incorporation into a methodology review on this topic [6, 12].

## Methods

This cost-effectiveness analysis is reported in line with the Consolidated Health Economic Evaluation Reporting Standards (CHEERS) statement [13]. Its aim was to compare the costs and effects of using each of four variant approaches, or ‘process models’ (i.e. workflows comprising a series of procedural stages, with underlying methods), to identify studies eligible for inclusion in a systematic review of the effects of undergraduate medical education in UK general practice settings. Methods and results of the ‘case study’ systematic review are reported elsewhere [14]. A brief summary of its search methods and study eligibility criteria is provided in Table 1.

**Table 1:** Summary of search methods and PICO eligibility criteria used in the ‘case study’ systematic review of the effects of undergraduate medical education in UK general practice settings [14]

Search methods	
• Electronic databases searched (from inception to March 2013): MEDLINE, Embase, Cumulative Index to Nursing and Allied Health Literature (CINAHL), PsycINFO, British Education Index (BEI), Education Resources Information Center (ERIC), and Australian Education Index (AEI). • Journals searched by hand (from January 1990 to March 2013): Medical Education, Family Medicine, and British Journal of General Practice. • Citation searches using references lists of eligible study reports identified using electronic and hand searches.	
Eligibility criteria	
• Population: undergraduate medical students in the UK. • Intervention: medical education delivered in a general practice setting (via student placement). • Comparator: medical education delivered in a hospital setting (via student placement). • Outcomes: cognitive, behavioural, emotional change or learning, perceived benefits and dis-benefits, costs. • Study designs: primary empirical research studies of any design, using quantitative and/or qualitative methods. • Other: studies published since 1990 and reported in English language.	

The cost-effectiveness analysis was conducted using a basic decision-analytic modelling framework. This involved the use of prospectively collected meta-data, on time use and eligibility (screening) decisions made by the ‘case study’ review team, to model the changes in flows of eligible and ineligible study records and full-text reports through each stage of the screening process that would have resulted from a decision to implement each process model, and thereby, to investigate differences in costs (resource use) and effects (recall) between the variant approaches (process models).

**Fig. 1:**
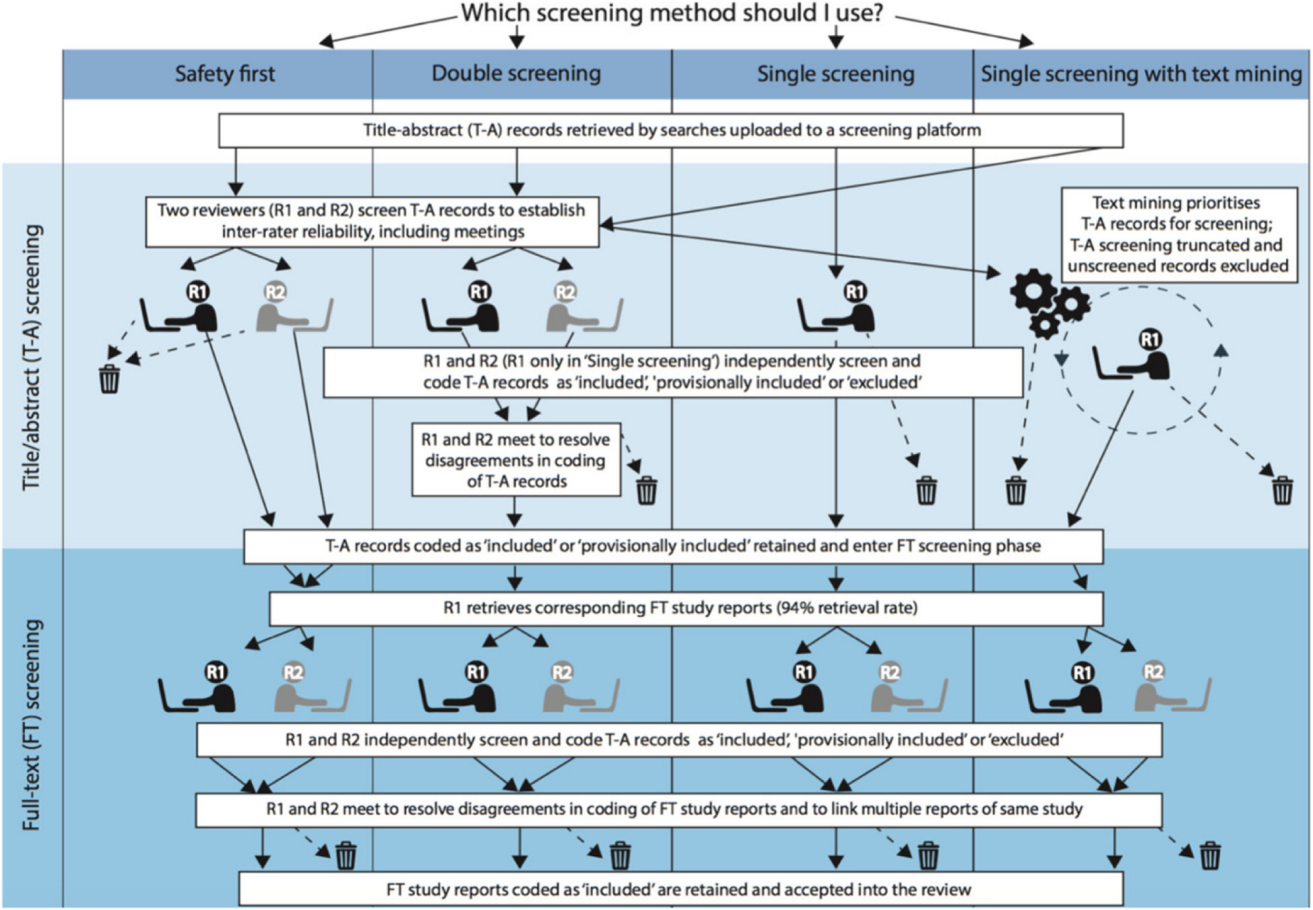
Four screening methods compared in the analysis

The structure of the decision-analytic model is a basic decision tree, as illustrated in Fig. 1. The decision node (i.e. a node representing a decision between the four variant approaches) is shown at the top of Fig. 1, and arrows represent the flow of title-abstract records and corresponding full-text study reports through the screening process in each process model. The four process models are described below. They differ only in those procedural steps highlighted in the upper portion (light blue-shaded area) of Fig. 1, which concern the management, screening and coding (against the review’s eligibility criteria) of title-abstract records—as described below. All procedural steps in the full-text screening stage (lower portion, dark blue-shaded area of Fig. 1) are identical between the four process models: once title-abstract screening is completed, those records classified as ‘included’ or ‘provisionally included’ are retained, corresponding full-text study reports are retrieved and all of these full-texts are manually screened by two reviewers working independently, who then meet to resolve disagreements in their application of study eligibility criteria and to link together multiple full-text reports of the same eligible study. In all modelled scenarios, full-text reports are coded as either ‘included’ or ‘excluded’. In the ‘case study’ review, reviewers in practice recorded one of eight hierarchical ‘excluded’ codes for each full-text report, each denoting a specific exclusion criterion (for example ‘excluded—not in the UK’, and ‘excluded—learning not in general practice’—see [14] for further details).

Since the objective of the study identification process in systematic reviews is to identify all those studies that would meet their pre-specified eligibility criteria, we operationalised the analytic unit of effect as ‘a citation saved from inappropriate exclusion’ (i.e. to reflect our strong aversion to excluding a record of a study that in fact meets eligibility criteria: a ‘false negative’), compared with the least effective model in terms of its recall. This analytic unit of effect can be viewed as a measure of the performance of each ‘process model’ (approach) in identifying eligible studies: its effectiveness.

The ‘double screening’ model was selected for investigation because it represents a set of recommended and commonly used procedures to identify and select eligible studies in Cochrane and other systematic reviews [9, 10]. However, the procedures applied in this approach are also ‘resource-hungry’ and, if there is high agreement between reviewers in their application of eligibility criteria, the cost per citation ‘saved’ from inappropriate exclusion—which can be viewed as a composite measure of the cost-effectiveness of each ‘process model’—may be high. Two of the other three ‘process models’ were selected for investigation because they are commonly used variants on a conventional ‘double screening’ approach. These can be viewed, respectively, as representing more (‘safety first’) and less (‘single screening’) cautious approaches to the title-abstract screening stage (see below). Finally, the ‘single screening with text mining’ model was selected because text mining has, in recent years, been advanced as a tool that can substantively reduce screening workload in systematic reviews; however, further evaluation is needed before it can be considered a reliable and widely accepted approach [15–17]. ‘Safety first’ was the method actually applied in the ‘case study’ systematic review. Each of the four variant approaches (process models) is described below.

### Safety first

The first step in the ‘safety first’ process model (as in all four approaches) is that all title-abstract records retrieved by electronic searches and other search methods are uploaded to a screening platform [18] and de-duplicated, with unique records entering the title-abstract screening stage. Next, two reviewers (R1 and R2) are allocated sequential batches of the same 100–200 title-abstract records for independent manual screening. In this preliminary stage of the process, screening of each batch is followed by a teleconference between the reviewers to discuss disagreements in their application of study eligibility criteria, with the aim of establishing a high level of inter-rater reliability in advance of the main tranche of title-abstract screening.

In the main tranche of title-abstract screening that follows, the two reviewers independently screen and assign one of three mutually exclusive codes to each of the remaining title-abstract records: ‘included’ (i.e. records clearly relevant to the review); ‘provisionally included’ (i.e. records of unclear relevance based on the title-abstract, including ‘title-only’ records with no abstract) or ‘excluded’ (i.e. records clearly irrelevant to the review, to be discarded). In the ‘case study’ review, reviewers in practice recorded one of eight hierarchical ‘excluded’ codes, each denoting a specific exclusion criterion (see [14] for further details).

The key feature of the ‘safety first’ approach is that a decision by either reviewer (R1 or R2) to assign an ‘included’ or ‘provisionally included’ code to a title-abstract record is taken as sufficient for that record to proceed into the full-text screening stage. In line with the ‘safety first’ process model implemented in the ‘case study’ systematic review, a decision by either reviewer to assign an ‘included’ or ‘provisionally included’ code to a title-abstract record also triggers immediate retrieval of the corresponding full-text study report (i.e. even if the other reviewer’s decision is to assign the ‘excluded’ code). One reviewer (R1) is assigned to obtain the corresponding full-text study report for each ‘included’ or ‘provisionally included’ record. Full-texts are retrieved in electronic copy, either online or from university library resources, or alternatively in hard copy via the university library or an inter-library loan. Next, the two reviewers (R1 and R2) again work independently to screen each full-text against eligibility criteria; however, in the full-text screening stage, eligibility (coding) disagreements are flagged in real time for immediate discussion and resolution between the two reviewers. This means that title-abstract and full-text screening stages are effectively conducted in parallel, with full-texts retrieved—and final eligibility decisions made and recorded—as soon as possible after either reviewer has coded a title-abstract record as ‘included’ or ‘provisionally included’. The latter represents a variation on common practice in systematic reviews, which conventionally involves conducting title-abstract and full-text screening stages in linear sequence (i.e. fully completing title-abstract screening before commencing full-text screening—see, for example, the ‘double screening’ process model, below).

‘Safety first’ can be viewed as a more cautious approach to title-abstract screening than conventional ‘double screening’ (described below) because it eliminates the possibility that reviewers might reach an incorrect consensus decision to exclude a title-abstract record of an eligible study prior to examining the corresponding full-text. However, it could also increase the forward flow of ‘false positive’ records (i.e. records of ultimately ineligible studies coded as ‘included’ or ‘provisionally included’ by one or both reviewers) into the full-text screening stage. As such, the net impacts of this approach on overall screening workload and associated costs are unclear. We note that some methods guidance suggests study eligibility should also be checked with the authors of each primary study [11], but we have not modelled this step in the current analysis.

### Double screening

‘Double screening’ was modelled as an identical set of procedures to those implemented in ‘safety first’, except that in this approach, both reviewers are required to agree to assign an ‘included’ or ‘provisionally included’ code to a title-abstract record before it is allowed to proceed to enter the full-text screening stage. The two reviewers (R1 and R2) therefore meet to discuss and resolve any disagreements between their independent title-abstract screening (coding) decisions, and make final consensus decisions on the eligibility of these title-abstracts, before corresponding full-texts are retrieved for examination. We modelled the latter procedural step using all those title-abstract records the two reviewers’ title-abstract coding decisions had disagreed about when using a ‘safety first’ approach in the ‘case study’ review.

### Single screening

‘Single screening’ was again modelled as an identical set of procedures to those implemented in ‘safety first’, except that only one reviewer (R1) is assigned to manually screen all retrieved title-abstract records against eligibility criteria, instead of two reviewers (R1 and R2) working independently. For costing, R1 was modelled as a research officer and R2 as a clinical academic (see below for details of costing methods); in practice, the individuals concerned are experienced systematic reviewers (see also the ‘Discussion’ section).

As well as corollary reductions in research staff time invested in title-abstract screening, the ‘single screening’ process model (as with the ‘safety first’ approach) eliminates the need for meetings to discuss and resolve coding disagreements. However, ‘single screening’ is also widely perceived as a less conservative approach compared with ‘safety first’ and ‘double screening’, because it relies on the judgement of a single person to apply eligibility criteria accurately and consistently, and therefore has the potential to increase the frequency of ‘false negative’ eligibility decisions (i.e. to reduce recall) [19], which could lead to syntheses based on incomplete sets of study data, with corollary risk of introducing study selection bias into the systematic review process and its findings.

### Single screening with text mining

The ‘single screening with text mining’ approach was modelled as an identical set of procedures to those implemented in the ‘single screening’ ‘process model’, except that text mining is used to prioritise title-abstract records for duplicate manual screening, and the screening process is truncated before all title-abstract records have been screened, with the remainder being automatically excluded from the review and discarded. In the current analysis, we modelled an ‘active learning’ scenario in which one reviewer (R1) commences title-abstract screening as usual and initially small sets of title-abstract records coded as ‘included or provisionally included’ or ‘excluded’ are used to train a classifier (a machine learning algorithm), which then automatically classifies all remaining (unscreened) records and returns an ordered list, with those records most likely to be eligible placed higher. The ‘active learning’ process continues in the simulation until all studies have been screened ‘manually’. We ran this simulation ten times, beginning with a random sample each time. We then assessed the consistency of results graphically and by examining the relative rank-order placement of citations across different ‘runs’ of the simulation. In the modelled scenario, the reviewer continues to screen records in prioritised order, the ‘active learning’ sequence is repeated (i.e. the classifier is re-trained and a new, re-ordered list is created) after every 25 title-abstract records have been screened, and title-abstract screening is truncated after a certain proportion of all title-records have been screened and coded, with all remaining records automatically excluded. Use of a ‘single screening with text mining’ approach can substantively reduce title-abstract screening workload, with corollary reductions in research staff time, needed to complete this stage. Current evaluations suggest that between 30 % and more than 90 % of workload might be reduced using this approach [16]; however—in addition to potential adverse effects of the ‘single screening’ approach, described above—adjunctive use of text mining could, when applied in this way, further reduce recall if the set of automatically excluded records includes ‘false negatives’ (i.e. records of eligible studies).

In order to determine a threshold recall rate to be modelled in the cost-effectiveness analysis, we conducted a retrospective simulation study to evaluate the performance of the ‘single screening with text mining’ approach, had this been implemented in the ‘case-study’ systematic review. Because simulation results showed that the use of text mining would invariably not have achieved 100 % recall in the ‘case study’ review until after the large majority of prioritised title-abstract records had been manually screened, a decision to deploy text mining in this review would in practice (and, as is typical [16]) have represented a trade-off between recall and workload. For the cost-effectiveness analysis, we therefore modelled a scenario in which the adjunctive use of text mining achieved 95 % recall, which (on average) occurred in simulations after 36 % of retrieved records had been manually screened. Our decision to model this scenario effectively meant we set ‘single screening with text mining’ to be the least effective among the four compared process models (i.e. at 95 %, it was set to achieve the lowest recall, which is used to calculate the number of citations ‘saved’ from inappropriate exclusion in the denominator of the cost-effectiveness equation).

We additionally investigated a further variant of each of the above four ‘process models’, in which the procedural step of classifying each title-abstract record *does not* incorporate the option of assigning a ‘provisionally included’ code instead of an ‘included’ or ‘excluded’ code. Many systematic reviews include a ‘provisionally included’ code option at the title-abstract screening stage for use to mark ‘tricky’ and/or ‘title-only’ records (i.e. those without an abstract) for later full-text assessment. While incorporating this code option provides a ‘safety net’ for reviewers when they are unsure about whether a record meets all eligibility criteria, it could increase overall screening workload by increasing the forward flow of ‘false positive records’ into the full-text screening stage (i.e. causing more corresponding full-text reports that do not ultimately meet eligibility criteria to be retrieved and unnecessarily examined). To simulate the impact of *excluding* this code option in each of the four variant ‘process models’ under investigation, we calculated the incremental costs associated with identifying each eligible study in each model based on the assumption that, in the absence of a ‘provisionally included’ code option, 50 % of those title-abstract records assigned this code that had an abstract would instead have been coded as ‘excluded’ and discarded, whereas the all title-only records would instead have been coded as ‘included’ (based on a ‘precautionary principle’). We also modelled a pair of simple, deterministic univariate analyses (5a and 5b in Table 5) in which the 50 % assumption concerning ‘provisionally included’ records with an abstract was varied +/− 25 % (i.e. 25 and 75 %).

Overall, this provided eight (4 × 2) variant process models for investigation in the cost-effectiveness analysis, each comprising variant sets of sequential procedural stages (see Fig. 1 and descriptions above). For the ‘single screening with text mining’ process model *with* a ‘provisionally included’ code option, simulations showed that, on average, this approach achieved 95 % recall after 39 % records had been manually screened.

The specific research objectives addressed by the cost-effectiveness analysis reported here were as follows:

1. To estimate the incremental costs (resource use) and effects (recall of studies included in the review) associated with the use of four variant approaches to title-abstract screening in the ‘case study’ systematic review; and
2. To estimate the incremental cost-effectiveness of using each approach, by combining estimates of incremental costs and effects.

The analytic perspective of the cost-effectiveness analysis was that of the systematic review author team’s research institution (a ‘single provider’ perspective). It therefore included the costs of those items of resource use expected to be the main drivers of differences between process models in costs—namely, differences in the quantities of research staff (reviewer) time allocated to identifying eligible studies, comprising time spent on manually screening title-abstract records and retrieving and examining full-text reports, and time spent in discussion to reach consensus on eligibility decisions, resulting from the different flows of study records and reports through each variant process model. The research team conducting the ‘case study’ systematic review had access to the large majority of full-text study reports via electronic library resources (online databases) provided by university subscription at no marginal cost per study report, so this item of resource use was not included in the costing.

To measure resource use in the ‘safety first’ process model (i.e. the method applied in the review), members of the ‘case study’ review team prospectively recorded the time allocated by each member of research staff to the completion of title-abstract and full-text screening, as well as the time allocated to full-text retrieval, and to discuss and resolve disagreements about the eligibility (coding) of full-text study reports. We then used these 'time use' data to estimate quantities of resource use associated with the procedural steps included within each process model (expressed in natural units, namely minutes of research staff time).

We next valued quantities of resource use by applying local unit costs obtained from university administrative database records that included details of the budget for this specific review project (this step involved simple multiplication of the relevant unit cost by the number of units of each included item of resource use: minutes of research staff time). Estimated unit costs of research staff time incorporated salaries, direct salary costs (such as national insurance and pension contributions) and university ‘indirect’ and ‘estates’ costs and were estimated separately for each of two categories of research staff involved in conducting the screening.

All costs are reported in 2013 UK GBP (£s)—the same price year and currency in which the reported costs were incurred. Estimated costs may therefore be considered specific to the UK higher education setting but, notably, they also incorporate ‘London weighting’ (i.e. an effective uplift in direct salary and university ‘indirect’ and ‘estates’ costs compared with universities located in other areas of the UK). For the ‘double screening’ model, the unit cost of resolving each disagreement about the eligibility (coding) of a title-abstract record by teleconference after the main tranche of title-abstract screening had been completed (see Fig. 1) was assumed to be the same as that of the same task undertaken for the purpose of establishing inter-rater reliability (see, for example, ‘safety first’, above).

All costs and effects incorporated into the cost-effectiveness analysis occurred within the time horizon of the screening process (i.e. from the start of the title-abstract screening stage to the end of the full-text screening stage) which was completed over a 19-week period during 2013 (and therefore no discount rate was applied). Cost-effectiveness was assessed in terms of the ‘incremental cost per citation saved from inappropriate exclusion’ (i.e. the incremental cost-effectiveness ratio, or ICER [20]) as a result of implementing each of the four variant study identification procedures (process models), compared with the least effective method in terms of its recall. This involved combining estimates of the incremental cost (resource use) with estimates of the incremental effect (the number of citations ‘saved’ from inappropriate exclusion) of using each of the variant process models, compared with the least effective model. Our decision to conduct a cost-effectiveness analysis reflects our interest in achieving a specified unit of output (i.e. a citation ‘saved’ from inappropriate exclusion) at the lowest cost in terms of resource use associated with this unit of output (effect).

We next conducted a series of simple, deterministic univariate sensitivity analyses to assess the resilience of our estimates of cost-effectiveness to plausible variations in the values of selected key input parameters, namely: time to screen a title-abstract record (+/− 50 %; sensitivity analysis 1a and 1b in Tables 4 and 5); time to screen a full-text study report (+/− 50 %; sensitivity analysis 2a and 2b in Tables 4 and 5); time to discuss and resolve a disagreement about the eligibility (coding) of a full-text study report (+/− 50 %; sensitivity analysis 3a and 3b in Tables 4 and 5); and unit costs (+/− 50 %; sensitivity analysis 4a and 4b in Tables 4 and 5). Finally, we investigated the impact of reduced recall on findings and conclusions of the case study review by qualitatively assessing the contribution to the ‘case study’ review of those studies that would have been excluded from consideration as a consequence of using each variant approach, if applicable.

## Results

### Overall impacts on workflows

**Fig. 2:**
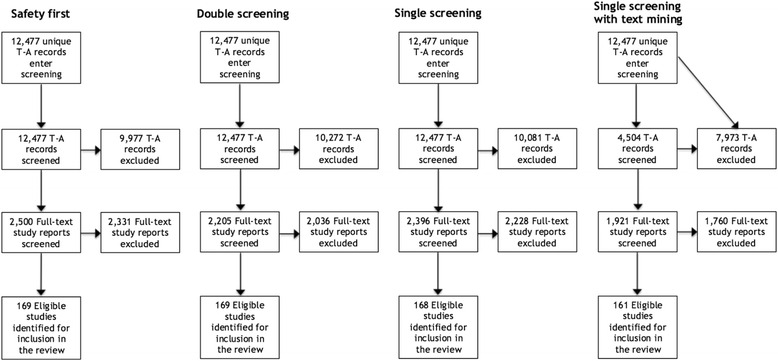
Modelled flows of records and study reports through screening, *with* a ‘provisionally included’ code

**Fig. 3:**
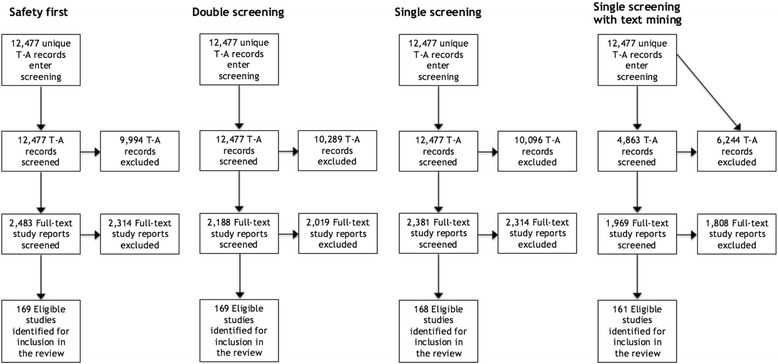
Modelled flows of records and study reports through screening, *without* a ‘provisionally included’ code

Figures 2 and 3 illustrate modelled flows of study records and corresponding full-text reports from the title-abstract screening stage into the full-text screening stage, culminating in studies being accepted into the review, and how these differ between each of the 4 × 2 variant process models, using PRISMA-style flow diagrams [21]. These figures illustrate differences in workload between the four approaches, as well as trade-offs between workload and recall. In particular, they illustrate the large modelled reduction in title-abstract screening workload of 64 % (*with* a ‘provisionally included’ code option) or 61 % (*without* a ‘provisionally included’ code option) associated with the use of the ‘single screening with text mining’—and corollary reductions in full-text screening workload—compared with each of the other three approaches (in which all title-abstract records are screened), set against the reduced recall of this approach (95 % compared with 99–100 %).

### Impacts on resource use, costs and cost-effectiveness

Table 2 shows estimated resource use per unit (as measured in the ‘case study’ systematic review), and Table 3 shows unit costs incorporated as data inputs into the cost-effectiveness analysis.

**Table 2:** Estimated resource use per unit: research staff time

Item	Estimated resource use per unit (minutes)
Time to screen a title-abstract record	1.0
Time to discuss and resolve a disagreement about the eligibility (coding) of a title-abstract record	5.0
Time to retrieve a full-text study report	4.0
Time to screen a full-text study report	5.0
Time to discuss and resolve a disagreement about the eligibility (coding) of a full-text study report	5.0

**Table 3:** Unit costs: research staff

Item	Unit cost (per minute)
Reviewer 1 (R1) time: research officer	£0.97
Reviewer 2 (R2) time: clinical academic	£1.38
Average (mean) unit cost of R1 and R2	£1.175

All costs are expressed in 2013 GBP (£) prices

#### With ‘provisionally included’ code option

Table 4 presents main results, including estimates of incremental resource use, costs, effects and cost-effectiveness associated with each process model for the four variants *with* a ‘provisionally included’ code option. ‘Single screening with text mining’ was set to be the least effective approach, in terms of recall, identifying 95 % of eligible study reports. Incremental results in Table 4 (and Table 5) are therefore presented in comparison to the ‘single screening with text mining’ approach.

**Table 4:** Incremental costs, effectiveness and cost-effectiveness (with ‘provisionally included’ code option)

	Safety first			Double screening			Single screening			Single screening with text mining		
Resource use and costs—research staff												
Resource use item	Time (min)	Unit cost (£)	Cost (£)	Time (min)	Unit cost (£)	Cost (£)	Time (min)	Unit cost (£)	Cost (£)	Time (min)	Unit cost (£)	Cost (£)
Teleconferences to establish inter-rater reliability	1200	1.175	1410	1200	1.175	1410	–	–	–	–	–	–
Title-abstract screening	24,954	1.175	29,321	24,954	1.175	29,321	12,477	0.97	12,103	4504	0.97	4369
Teleconferences to resolve disagreements about title-abstract eligibility (coding) decisions	–	–	–	5420	1.175	6371	–	–	–	–	–	–
Full-text retrieval	10,000	0.97	9700	8820	0.97	8555	9584	0.97	9296	7684	0.97	7453
Full-text screening	25,000	1.175	29,375	22,050	1.175	26,016	23,960	1.175	28,153	19,210	1.175	22,572
Teleconferences to resolve disagreements about full-text eligibility (coding) decisions	2950	1.175	3466	2950	1.175	3466	2950	1.175	3466	2950	1.175	3466
Total cost	£73,272			£75,139			£53,018			£37,860		
*Incremental cost*	*£35,412*			*£37,279*			£15,158			–		
Effectiveness—number of inappropriate exclusions avoided												
Recall	100 %			100 %			99 %			95 %		
Number of eligible studies identified	169			169			168			161		
*Incremental effectiveness*	*8*			*8*			*7*			–		
Cost-effectiveness—incremental cost per inappropriate exclusion avoided (ICER)												
*Base case*	*£4427*			*£4660* ^*a*^			*£2165*			–		
Sensitivity analysis 1a	£2867			£3100^a^			£1613			–		
Sensitivity analysis 1b	£5986			£6219^a^			£2718			–		
Sensitivity analysis 2a	£4008			£4438^a^			£1767			–		
Sensitivity analysis 2b	£4852			£4855^a^			£2564			–		
Sensitivity analysis 3a	£4427			£4262^b^			£2358			–		
Sensitivity analysis 3b	£4427			£5058^a^			£2358			–		
Sensitivity analysis 4a	£2213			£2330^a^			£1179			–		
Sensitivity analysis 4b	£4274			£4624^a^			£832			–		

All costs are expressed in 2013 GBP (£) prices

^a^Dominated by ‘safety first’

^b^Dominates ‘safety first’

The italics highlight rows containing the principal results

**Table 5:** Incremental costs, effectiveness and cost-effectiveness (without ‘provisionally included’ code option)

	Safety first			Double screening			Single screening			Single screening with text mining		
Resource use and costs—research staff												
Resource use item	Time (min)	Unit cost (£)	Cost (£)	Time (min)	Unit cost (£)	Cost (£)	Time (min)	Unit cost (£)	Cost (£)	Time (min)	Unit cost (£)	Cost (£)
Teleconferences to establish inter-rater reliability	1200	1.175	1410	1200	1.175	1410	–	–	–	–	–	–
Title-abstract screening	24,954	1.175	29,321	24,954	1.175	29,321	12,477	0.97	12,103	4863	0.97	4717
Teleconferences to resolve disagreements about title-abstract eligibility (coding) decisions	–	–	–	5400	1.175	6345	–	–	–	–	–	–
Full-text retrieval	9932	0.97	9634	8752	0.97	8489	9524	0.97	9238	7876	0.97	7640
Full-text screening	24,830	1.175	29,175	21,880	1.175	25,709	23,810	1.175	27,977	19,690	1.175	23,136
Teleconferences to resolve disagreements about full-text eligibility (coding) decisions	2950	1.175	3466	2950	1.175	3466	2950	1.175	3466	2950	1.175	3466
Total cost	£73,006			£74,740			£52,784			£38,959		
*Incremental cost*	*£34,047*			*£35,781*			£13,825			–		
Effectiveness—number of inappropriate exclusions avoided												
Recall	100 %			100 %			99 %			95 %		
Number of eligible studies identified	169			169			168			161		
*Incremental effectiveness*	*8*			*8*			*7*			–		
Cost-effectiveness—incremental cost per inappropriate exclusion avoided (ICER)												
*Base case*	*£4256*			*£4473* ^*a*^			*£1975*			–		
Sensitivity analysis 1a	£2718			£2935^a^			£1447			–		
Sensitivity analysis 1b	£5794			£6010^a^			£2502			–		
Sensitivity analysis 2a	£3878			£4187^a^			£1629			–		
Sensitivity analysis 2b	£4633			£4634^a^			£2321			–		
Sensitivity analysis 3a	£4256			£4076^b^			£1975			–		
Sensitivity analysis 3b	£4278			£4892^a^			£2001			–		
Sensitivity analysis 4a	£2128			£2236^a^			£987			–		
Sensitivity analysis 4b	£6384			£6709^a^			£2962			–		
Sensitivity analysis 5a	£4276			£4493^a^			£1998			–		
Sensitivity analysis 5b	£4287			£4504^a^			£2013			–		

All costs are expressed in 20XX GBP (£) prices

^a^Dominated by ‘safety first’

^b^Dominates ‘safety first’

The italics highlight rows containing the principal results

Compared with ‘single screening with text mining’, the ‘single screening’ approach ‘saved’ seven citations (study records/reports) from inappropriate exclusion (99 % recall), while ‘safety first’ and ‘double screening’ each ‘saved’ eight citations (100 % recall)—these were the two most effective approaches. However, in the base case analysis, the ‘single screening with text mining’ approach was also the least costly to implement (Table 4; modelled *with* a ‘provisionally included’ code option), at an estimated total cost of £37,860 (i.e. adding together costs incurred in both the title-abstract and full-text screening stages), with a higher total cost associated with implementation of ‘single screening’ (40 % higher), ‘safety first’ (94 % higher) and ‘double screening’ (98 % higher), the latter being the most costly approach at a total cost of £75,139.

Compared with ‘single screening with text mining’ (set to 95 % recall), estimated incremental cost-effectiveness ratios (ICERs) (i.e. incremental cost per citation ‘saved’ from inappropriate exclusion) for ‘double screening’ (100 % recall) and ‘safety first’ (100 % recall) were £4660 and £4427, respectively (base case analysis). As such, the ‘double screening’ approach was dominated by ‘safety first’ in terms of cost-effectiveness (i.e. ‘double screening’ and ‘safety first’ were equally effective but ‘double screening’ was more costly). Compared with ‘single screening with text-mining’, the ICER for ‘single screening’ (99 % recall) was £2165 per citation ‘saved’ from inappropriate exclusion (base case analysis).

In sensitivity analyses, ranges of estimated ICERs (compared with ‘single screening with text mining’) were £2213 to £5986 per inappropriate exclusion avoided for ‘safety first’ approach, £2330 to £6219 for ‘double screening’ and £832 to £2718 for ‘single screening’. Within each sensitivity analysis, patterns of results for incremental costs and effects between approaches were almost invariably consistent with the base case analysis. The exception was that, in the sensitivity analysis in which the resource input (staff time) allocated to meetings held to resolve ‘coding’ disagreements was reduced by 50 %, the ‘double screening’ approach dominated ‘safety first’; ‘double screening’ was equally effective but, in this case only, cost less than ‘safety first’. This result was observed because meetings to discuss and resolve disagreements about title-abstract records are required by the ‘double screening’ approach, but not by the ‘safety first’ approach. This result implies that the incremental costs of these two approaches are likely to be sensitive to amounts of time spent discussing and resolving coding disagreements.

#### Without a ‘provisionally included’ code option

Table 5 presents comparable results for the four process model variants *without* a ‘provisionally included’ code option. In the base case analysis, estimates of the incremental costs and cost-effectiveness of ‘double screening’, ‘safety first’ and ‘single screening’ (compared with ‘single screening with text mining’) were invariably lower than was found *with* a ‘provisionally included’ code option, driven largely by a marginal improvement in the simulated performance of text mining, which can be attributed to lower numbers of title-abstract records of ineligible studies being present among the set of ‘included or provisionally included’ records on which the classifier is iteratively trained when a ‘provisionally included’ code is not available.

In sensitivity analyses, ranges of estimated ICERs (compared with ‘single screening with text mining’) were £2128 to £6384 per inappropriate exclusion avoided for ‘safety first’ approach, £2236 to £6709 for ‘double screening’ and £987 to £2962 for ‘single screening’. Within each sensitivity analysis, patterns of results for incremental costs and effects between approaches were entirely consistent with those reported above from sensitivity analyses for variants of process models *with* the provisional include option. Results of the two additional sensitivity analyses conducted for variants of process models *without* the provisional include option (5a and 5b in Table 5), concerning our base case assumption of a 50 % exclusion rate among title-abstract records coded in practice as ‘provisionally included’ *with abstracts* (see the ‘Methods’ section), showed estimates of incremental cost-effectiveness were insensitive to a +/− 25 % variation in the exclusion rate among those records.

### Impact of reduced recall on the ‘case study’ review

As shown in Figs. 2 and 3 (and in Tables 4 and 5) above, the use of a ‘single screening’ approach would have resulted in the exclusion of one eligible study [22] from the ‘case study’ systematic review, while use of the ‘single screening with text mining’ approach would have resulted in the exclusion of eight other eligible studies [23–30]. Analysis of the contributions made by these nine ‘false negative’ studies to the ‘case study’ review found that all nine contributed only to the descriptive component of the review (i.e. were used to inform a descriptive summary of the included studies) but none were cited in relation to specific points of analysis within this component. None of these ‘false negative’ studies were among the set of studies incorporated into either the quantitative in-depth analysis, nor among the set incorporated into the in-depth qualitative synthesis (meta-ethnography). While one of the ‘false negative’ studies did provide a distinctive perspective concerning the influence of workplace-based learning in general practice on patient care [26], we believe this study would have been identified by one of the two complementary search methods deployed in the ‘case study’ review (namely, stakeholder consultation; the other complementary search method used, namely backward citation tracking [31, 32], would not have identified this study as it was not cited in reference lists of studies incorporated into the in-depth syntheses). These results indicate that there would have been negligible impact on the findings or conclusions of this ‘case study’ review as a consequence of reduced recall associated with use of ‘single screening’, or ‘single screening with text mining’, rather than the ‘safety first’ approach implemented in practice or conventional ‘double screening’.

## Discussion

### Summary of main findings

A first key finding from this analysis was that, in a systematic review of the effects of undergraduate medical education in UK general practice settings, the use of a ‘safety first’ approach to title-abstract screening—in which a record marked as ‘included’ (or ‘included or provisionally included’) by *any* reviewer ‘automatically’ proceeds to the full-text screening stage—was almost invariably equally effective and less costly than conventional ‘double screening’ (i.e. ‘safety first’ dominated ‘double screening’ in terms of cost-effectiveness). If this key finding was replicated in similar analyses of other systematic review datasets, conducted using a comparable modelling framework, this would justify the adoption of a ‘safety first’ approach for title-abstract screening in reviews that require broad and/or highly sensitive searches, on efficiency grounds. However, the results of the current study also highlighted that the relative efficiency of these two (and other) approaches is likely to vary between systematic reviews, contingent not only on the amount of time spent discussing and resolving coding disagreements in the title-abstract screening stage (as implied by the results of sensitivity analyses) but also on factors such as search yield (i.e. the total number of title-abstract records retrieved by searches), the inclusion rate among retrieved records, levels of topic expertise and experience among the reviewers and inter-reviewer reliability. For example, further investigation of ‘case study’ review data indicated that marginal efficiency gains from using a ‘safety first’ (compared with ‘double screening’) would have increased if larger numbers of title-abstract records had needed to be screened. Similarly, in the current ‘case study’ systematic review of undergraduate medical education in UK general practice settings, screening was completed by a medical student and GP academics, reflecting levels of expertise and familiarity with the topic that may not pertain in other reviews. Further research could therefore usefully include a focus on developing a better understanding of how variation in these factors may drive the incremental costs and effects of using ‘safety first’, compared with ‘double screening’ (or other approaches).

A second key finding was that, with recall set to 95 %, the use of ‘single screening with text mining’ would have resulted in overall title-abstract screening workload reductions (base case analysis) of 64 % (*with* a ‘provisionally included’ code option) or 61 % (*without* a ‘provisionally included’ code option), compared with each of the other approaches, and would therefore have incurred around half of the total cost of ‘safety first’ and ‘double screening’ (with these incremental costs being lower when comparisons were modelled *without* the ‘provisionally included’ code option, due to the improved performance of text mining in this scenario). This finding suggests that conducting electronic searches, then using text mining as an adjunct to a ‘single screening’ approach and applying a reasonable ‘stopping rule’ to truncate title-abstract screening, combined with complementary search methods, may represent a pragmatic and efficient approach to identifying eligible studies in large-scale, complex systematic reviews. However, this finding also highlights that decisions to use text mining as an adjunct to a ‘single screening’, ‘safety first’ or conventional ‘double screening’ approach, to prioritise records for manual screening, will be contingent on contextual factors, including the resources available to be allocated to title-abstract screening and the willingness of review teams and funders to sacrifice recall in order to substantively reduce the overall workload and total costs of systematic review production. The estimated ICERs from base case analyses of the two most conservative scenarios, ranging from £3158 (‘safety first’ with a ‘provisionally included’ code) to £4457 (‘double screening’ without a ‘provisionally included’ code) per citation ‘saved’ from inappropriate exclusion, further illustrate this trade-off. We further note that a similar trade-off would have applied in the current ‘case study’ review to a choice between the ‘single screening’ model and either of the two more costly, but also slightly more effective, approaches to title-abstract screening.

A third key finding was that incorporating a ‘provisionally included’ code option incurred higher resource use and associated costs in all four process models, due to consequent increases in the forward flow of ultimately ineligible (i.e. false positive) study records and reports into to the full-text screening stage, and would therefore have represented a less efficient strategy compared with excluding this code option.

### Limitations of the cost-effectiveness analysis

This cost-effectiveness analysis contributes a single study dataset to an emerging evidence base for the relative efficiency of variant approaches to title-abstract screening in systematic reviews. As described above, it was based on data prospectively collected alongside a ‘case study’ systematic review of the effects of undergraduate medical education in UK general practice settings, conducted by an experienced team of systematic reviewers with substantial experience in primary care and medical education research, and access to UK university infrastructure (e.g. extensive electronic library resources, and systematic review software that enabled concurrent, multi-user workflows to be implemented in the study identification stage of the ‘case study’ review). It is important to highlight that contextual factors such as these determine *absolute* levels of resource use associated with each of the four modelled approaches. Estimates of resource use (researcher time) and costs of study identification are also specific to design features of the ‘case study’ systematic review, for example, the number and complexity of criteria that needed to be applied to reach eligibility decisions and the complexity of the topic under review. As such, the generalisability of the findings of this cost-effectiveness analysis beyond the current ‘case study’ review, and to research settings other than experienced teams based in UK higher education institutions located in London, remains to be established. This empirical question can be addressed by conducting similar cost-effectiveness analyses using the simple modelling framework demonstrated in this article, in order to contribute to building an evidence base to help inform guidance on study data identification methods in systematic reviews.

The ideal primary study design for use as a framework for an economic evaluation to assess the cost-effectiveness of variant approaches to title-abstract screening would be an adequately powered cluster randomised controlled trial, in which a sample of review teams were randomly assigned to undertake screening for the same systematic review using each variant approach (‘process model’). While such studies are in principle possible, they are unlikely in practice due to the duplication of effort such a study design would entail and the corollary impact on costs of the research. In these circumstances, simple, model-based economic evaluations using single systematic review datasets offer a feasible, low-cost alternative that can help to build the evidence base. With improved electronic curation of systematic review meta-data, coupled with prospective recording of time use among review teams, we can amass the datasets needed for such analyses relatively quickly. This includes new and existing datasets produced as a by-product of the increasing number of reviews that use text mining in their screening workflows to support study identification [16]; such datasets need to be analysed to inform the further diffusion and use of this technology [17].

It is also important to highlight that the retrospective simulations of text mining performance used as the basis for modelling the flow of study records and reports through the ‘single screening with text mining’ process model cannot, by definition, be conducted until after the screening and study selection process has been completed. As such, these data are not available to review teams in advance, to inform a decision about whether or not to use text mining, which needs to be taken at the protocol stage. This consideration highlights that, in practice, decisions to deploy text mining in the way described (i.e. to prioritise records for manual screening) are currently made on pragmatic grounds (for example, the resource available to be allocated to screening in relation to the total number of records that need to be screened) but also that such decisions should be made cognisant of evidence for the potential trade-off between reduced screening workload (cost) and reduced recall (effectiveness). Similarly, the analysis presented in this article assumes that those studies identified for inclusion in the ‘case study’ systematic review represent a complete, ‘gold standard’ reference set of all eligible studies (and recall is measured against this standard); however, in practice, this is not known at either the outset or end of any review, so authors cannot base pre-specified ‘stopping rules’ for truncating title-abstract screening on such data. Instead, pre-specified ‘stopping rules’ currently need to be formulated based on estimates of the predicted number of eligible title-abstract records (‘baseline inclusion rate’) among retrieved title-abstract records, based on preliminary screening of a random sample of those records [33]. The results of this analysis can, in conjunction with those of similar retrospective simulations of text mining performance in other systematic review datasets, be used to inform evidence-based guidance on ‘stopping rules’ for truncating title-abstract screening once a ‘sufficient’ proportion of prioritised title-abstract records have been manually screened, in order to provide ‘adequate’ insurance against the risk of ‘distorted assembly of data’ due to reviews potentially being based on less-than-complete sets of study data. The latter risk can also be mitigated in systematic reviews by the use of complementary search methods, such as backward and forward citation tracking, grey literature searches [10, 31, 32] and stakeholder consultation, in conjunction with electronic searches of the kind modelled in the current study.

## Conclusions

This study has demonstrated the application of a simple, model-based economic evaluation framework to assess the incremental costs, effects and cost-effectiveness of variant approaches to study identification in systematic reviews. Its key findings suggest that alternatives to the conventional ‘double screening’ approach, implemented without a ‘provisional include’ code option and integrating text mining, may warrant further consideration as promising, potentially more efficient approaches to identifying eligible studies for systematic reviews. Further, comparable economic evaluations of other systematic review datasets are needed to determine the generalisabiity of these findings to other systematic reviews and research settings, and also to help build an evidence base to inform updated guidance for review authors on study identification methods.
